# Defective natural killer cell anti-viral capacity in paediatric HBV infection

**DOI:** 10.1111/cei.12470

**Published:** 2015-02-16

**Authors:** I L Heiberg, L J Pallett, T N Winther, B Høgh, M K Maini, D Peppa

**Affiliations:** * Department of Paediatrics, Hvidovre Hospital, University of CopenhagenCopenhagen, Denmark; † Division of Infection and Immunity, UCLLondon, UK

**Keywords:** NK cells, anti-viral function, paediatric HBV infection, IFN-γ, NKp30

## Abstract

Natural killer (NK) cells exhibit dysregulated effector function in adult chronic hepatitis B virus (HBV) infection (CHB), which may contribute to virus persistence. The role of NK cells in children infected perinatally with HBV is less studied. Access to a unique cohort enabled the cross-sectional evaluation of NK cell frequency, phenotype and function in HBV-infected children relative to uninfected children. We observed a selective defect in NK cell interferon (IFN)-γ production, with conserved cytolytic function, mirroring the functional dichotomy observed in adult infection. Reduced expression of NKp30 on NK cells suggests a role of impaired NK-dendritic cell (DC) cellular interactions as a potential mechanism leading to reduced IFN-γ production. The finding that NK cells are already defective in paediatric CHB, albeit less extensively than in adult CHB, has potential implications for the timing of anti-viral therapy aiming to restore immune control.

## Introduction

Perinatal transmission of hepatitis B virus (HBV) is the most common mode of transmission world-wide. Despite the availability of an effective prophylactic vaccine, HBV infection during infancy or early childhood is common in areas of high endemicity. In these regions, mother-to-infant transmission accounts for approximately 50% of chronic infections. The age at infection primarily determines the rate of progression from acute to chronic infection, which is approximately 90% in the perinatal period, 20–50% in children aged 1–5 years and less than 5% in adults 1 2–4. Although the majority of children with chronic hepatitis B infection (CHB) are asymptomatic, they are at increased risk of developing progressive liver disease and complications such as hepatocellular carcinoma (HCC) before the third decade of life 5,6.

Perinatally infected children are considered predominantly to be in the immunotolerant phase, with a high viral load, detectable HBeAg and, initially, minimal detectable liver inflammation 4. However, our knowledge of the natural history of paediatric HBV infection is severely limited by the difficulty in sampling this group and age-matched controls. This has led to the widely held view that immunotolerant children are unlikely to respond to anti-viral therapy. Recent work in young adults in the immunotolerant phase of CHB challenge the dogma that younger patients lack an HBV-specific adaptive immune response, suggesting that they may be more suitable treatment candidates than considered previously 7–9. Thus, a more comprehensive understanding of the various immunological parameters during paediatric HBV infection may inform clinical management more clearly. In this study we have focused on natural killer (NK) cells, a critical component of anti-viral defence, enriched in the liver and shown to be dysregulated in adult CHB.

NK cells display a multitude of anti-viral effector functions through the production of cytokines and direct target cell recognition and lysis 10. The intensity and quality of NK cell cytotoxic and cytokine responses is regulated by a wide array of NK cells receptors that are finely tuned to ensure self-tolerance while permitting effective responses against invading pathogens 10,11. NK cell function may be calibrated further by the local cytokine microenvironment and bi-directional interactions between innate and adaptive immune cells 12. Besides their direct anti-viral function, it is recognized increasingly that NK cells also have an important immunoregulatory role 13–16.

Reports on the role of NK cells in HBV infection have focused on adult life, when chronic infection has usually already been long-established. We, and others, have previously demonstrated a functional dichotomy, with conserved NK cytolytic function and impaired IFN-γ, in adult patients with CHB 17–19. More recently we have shown that NK cell activity can regulate the adaptive immune response, as well as the degree of immunopathology, in CHB 16,20. Moreover, treatment with pegylated (Peg)-interferon (IFN)-α mediated functional augmentation of NK cell effector function, which correlated with peak virological responses, highlighting the anti-viral potential of NK cells 21. Little is known, however, about NK cell function in children with CHB. Previous studies have shown that neonatal NK cells have an immature phenotype that may compromise their capacity to respond to viral infections 22–25. It remains unknown how NK cell populations and functions are modified during the course of CHB from childhood to adulthood. Here we used multi-parametric flow cytometry to investigate whether the frequency, phenotype and function of NK cells is altered in a cohort of perinatally HBV-infected children and compared them to uninfected children.

## Materials and methods

### Study population

Eighteen children with CHB were included into the study. The patients were recruited from the Department of Paediatrics, Hvidovre Hospital, University of Copenhagen, Denmark. All patients were negative for HIV, hepatitis A, hepatitis C and hepatitis D virus and were considered healthy apart from their CHB. None of the children had received anti-viral treatment for HBV. Blood samples were obtained at the children's routine clinical visits during the period October 2008–August 2011. Sixteen healthy, HBsAg-negative children were included as controls during the period August 2010–August 2011. Patient characteristics and classification are summarized in Table 1. The study protocol was approved by the Ethical Committee Capital Region of Denmark, reference number H-KF-255584, and parents of all participants gave written informed consent.

**Table 1:** Patient characteristics

	Healthy controls	CHB individuals		
		*Active	**Inactive	§Immunotolerant
	*n* = 16	*n* = 5	*n* = 5	*n* = 8
Age, years: mean (± s.d.)	8 (± 3)	12 (± 4)	12 (± 4)	10 (± 4)
Range	4–15	5–16	6–17	4–15
Gender (F = female, M = male)	F = 5, M = 11	F = 4, M = 1	F = 2, M = 3	F = 5, M = 3
Race	Asian=0, African=2, Caucasian=14	Asian=2, African=2, Caucasian=1	Asian=1, African=2, Caucasian=2	Asian=7, Caucasian=1
ALT, IU/l: mean (± s.d.)	14 (± 5)	88·8 (± 42)	23·2 (± 8·9)	29·5 (± 7·4)
Range	6–25	61–164	14–37	18–36
Acquisition of HBV	n.a.	Vertically=1, horizontally=2, not traceable=2	Vertically=2, horizontally=1, not traceable=2	Vertically=1, not traceable=7
Viral load IU/ml: mean (± s.d.)	n.a.	9·4 × 10∧8 (± 1·7 × 10∧8)	535 (± 712)	3·2 × 10^8^ (± 2·8 × 10^8^)
Range		2000–3·6 × 10∧9	100–1600	1 × 10^6^–8·1 × 10^8^
HBeAg^+^	n.a.	4/5	0/5	8/8
Genotype	n.a.	B = 1, D = 2, E = 2	D = 2, E = 2, unknown=1	B = 6, D = 1, unknown=1

Patients with chronic hepatitis B (CHB) were analysed according to disease activity.

* Patients with active disease [hepatitis B e antigen (HBeAg^+/−^)] are characterized by elevated level of alanine transaminase (ALT) (>60) and elevated viral load (>2000 IU/ml).

** Patients in the inactive phase are HBeAg^−^, have low viral load (<2000 IU/ml) and ALT in the normal range.

§ Patients classified as immunotolerant are HBeAg^+^ and have high viral load (>1 million IU/ml) and ALT in the normal range; n.a. = not applicable; s.d. = standard deviation; HBV – hepatitis B virus.

### Isolation and storage of peripheral blood mononuclear cells (PBMCs) and plasma

Blood was obtained in 8-ml BD Vacutainer™ CPT ™ tubes with sodium heparin, and peripheral blood mononuclear cells (PBMCs) were isolated according to the manufacturer's instructions. Freezing medium [10% dimethylsulphoxide (DMSO) and 90% fetal calf serum (FCS)] was added, and the cells were stored at −135°C. Plasma was collected and frozen at −80°C.

### Extracellular staining and flow cytometric analysis

For phenotypical analysis, PBMCs isolated from children with CHB and uninfected controls were stained with fluorochrome-conjugated antibodies to CD3-phycoerythrin (PE)-cyanin 7 (Cy7), NK group 2D (NKG2D)-PE (eBioscience, Hatfield, UK), CD56-energy-coupled dye (ECD) (Beckman Coulter, High Wycombe, UK), tumour necrosis factor (TNF)-related apoptosis-inducing ligand (TRAIL)-PE, CD16-allophycocyanin (APC)-Cy7, CD94-fluorescein isothiocyanate (FITC), human leucocyte antigen D-related (HLA-DR)-V500, NKp46-V450 (BD Biosciences, Oxford, UK), NKG2A-Alexa700, NKG2C-PerCP (R&D Systems, Abingdon, UK) and NKp30-APC (Miltenyi Biotec, Surrey, UK) or isotype-matched controls, in the presence of fixable live/dead stain (Invitrogen, Paisley, Scotland, UK). Cells were acquired on a fluorescence activated cell sorter (FACS) LSRII multi-colour flow cytometer and analysed using FlowJo analysis software (Tree Star, Ashland, OR, USA).

### Cytokine production

For intracellular staining for IFN-γ production, PBMCs were incubated with 50 ng/ml of rhIL-12 (Miltenyi Biotec) and 50 ng/ml of rh-IL18 (R&D Systems) for 21 h at 37°C. One mM monensin (Sigma-Aldrich, Gillingham, UK) was added for the final 3 h. Cells were stained with antibodies to CD3-PE-Cy7 (eBioscience), CD16-APC-Cy7 (BD Biosciences) and CD56-ECD (Beckman Coulter) and subsequently fixed and permeabilized, followed by intracellular staining for IFN-γ-V450 (BD Biosciences). Dead cells were excluded by live/dead stain (Invitrogen).

For TNF-α production, PBMCs were stimulated with phorbol myristate acetate (PMA) (3 ng/ml) and ionomycin (100 ng/ml) (Sigma-Aldrich) for 3 h in the presence of 1 mM of monensin (Sigma-Aldrich). Cells were stained with antibodies to CD3-PE-Cy7 (eBioscience), CD16-APC-Cy7 (BD Biosciences) and CD56-ECD (Beckman Coulter) in the presence of live/dead stain followed by fixing, permeabilization and intracellular staining for TNF-α-FITC (BD Biosciences).

### CD107 degranulation assay

NK cell degranulation was measured as described previously 26. Briefly, PBMCs were incubated with K562 cells [5:1 effector : target (E : T) ratio] for 3 h at 37°C following overnight stimulation with a combination of 50 ng/ml recombinant human (rh)IL-12 and rhIL-18 (Miltenyi Biotec, R&D Systems). CD107a-PE antibody (BD Biosciences) was added at the time of stimulation with target cells along with 1 mM monensin prior to staining and acquisition.

### Plasma concentration of cytokines and chemokines determined by cytometric bead array (CBA)

CBA 11 plex RTU FlowCytomix Kit (eBioscience) was used for the determination of IL-1b, IL-2, IL-4, IL-5, IL-6, IL-8, IL-10, IL-12p70, IFN-α, IFN-γ, TNF-α, TNF-β, chemokine (C-C motif) ligand 3 (CCL3), chemokine (C-X-C motif) ligand 9 (CXCL9) and chemokine (C-X-C motif) ligand 10 (CXCL10) in plasma samples, according to the manufacturers' protocols.

### Statistical analysis

Statistical analysis was performed between HBV patients and healthy controls using the Mann–Whitney *U*-test. Correlations between variables were evaluated with the Spearman's rank correlation test (Prism version 4; GraphPad Software Inc., San Diego, CA, USA). *P* < 0·05 was considered to be significant for all tests.

## Results

### Comparable NK cell frequency and subsets in CHB and healthy children

**Fig 1:**
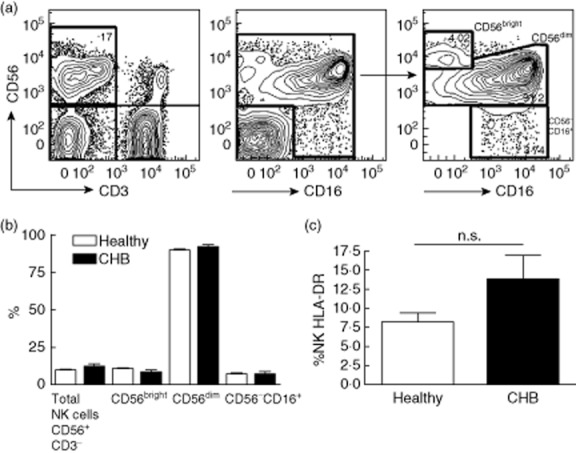
Comparable distribution of natural killer (NK) cell subsets between children with chronic hepatitis B (CHB) and uninfected healthy controls. Phenotypical analysis was performed on isolated peripheral blood mononuclear cells (PBMCs) by multi-colour flow cytometry. (a) Representative contour plots from a child with CHB are shown, gating on CD56^+^CD3^−^ PBMCs to identify NK cells and CD56^+^CD16^+^ to help identify the CD56^bright^, CD56^dim^ and CD56^–^CD16^+^ NK cell subsets. (b) Summary bar charts of proportions of total NK cells and subsets, CD56^bright^, CD56^dim^ NK cells and CD56^−^CD16^+^ NK cells in children with CHB (*n* = 18) and healthy children (*n* = 16). (c) Summary data comparing expression of human leucocyte antigen D-related (HLA-DR) on NK cells in children with CHB (*n* = 18) and healthy children (*n* = 16). Results are expressed as mean ± standard error of the mean.

NK cell populations exhibit dynamic changes during the first years of life and become more comparable with adult NK cells after the age of 5 years 27. To assess the impact of HBV infection on NK cell frequency and subset distribution we compared the frequency and distribution of different NK cell subpopulations (CD56^bright^ and CD56^dim^) in the peripheral blood of 18 children with CHB and 16 healthy children (Table 1). We found no significant difference in the proportions of total circulating NK cells (CD56^+^CD3^−^) or CD56^bright^ CD16^−/dim^, CD56^dim^ CD16^+^ and CD56^–^CD16^+^ NK cell subsets between the two groups (Fig. 1a,b). To evaluate whether there were any perturbations in the proportions of NK cell subsets according to disease activity, results were analysed according to HBeAg status, viral load and levels of alanine transaminase (ALT). Our data demonstrate that there was no skewing of frequencies within the NK cell peripheral compartment during paediatric HBV infection, irrespective of disease status (data not shown). Similarly, we did not detect any significant differences in frequencies of CD56^+^CD3^+^ T cells, previously noted to be increased in cytomegalovirus (CMV)-seropositive individuals 28, between the two groups [mean ± standard error of the mean (s.e.m.), 2·291 ± 0·4768 *versus* 2·209 ± 0·5741, *P* = 0·8968, data not shown].

The expression of HLA-DR on NK cells has been used previously as a marker of cellular activation 29. We therefore analysed the levels of the activation marker HLA-DR on peripheral NK cells and subsets in CHB children and healthy controls. Expression of HLA-DR on NK cells was not found to be statistically different between CHB children and healthy controls (Fig. 1c).

### Impaired IFN-γ production but intact cytolytic function by NK cells in paediatric HBV patients

Given that NK cells have comparable subset distribution and levels of activation between the two groups, we next assessed the effector function of these cells.

**Fig 2:**
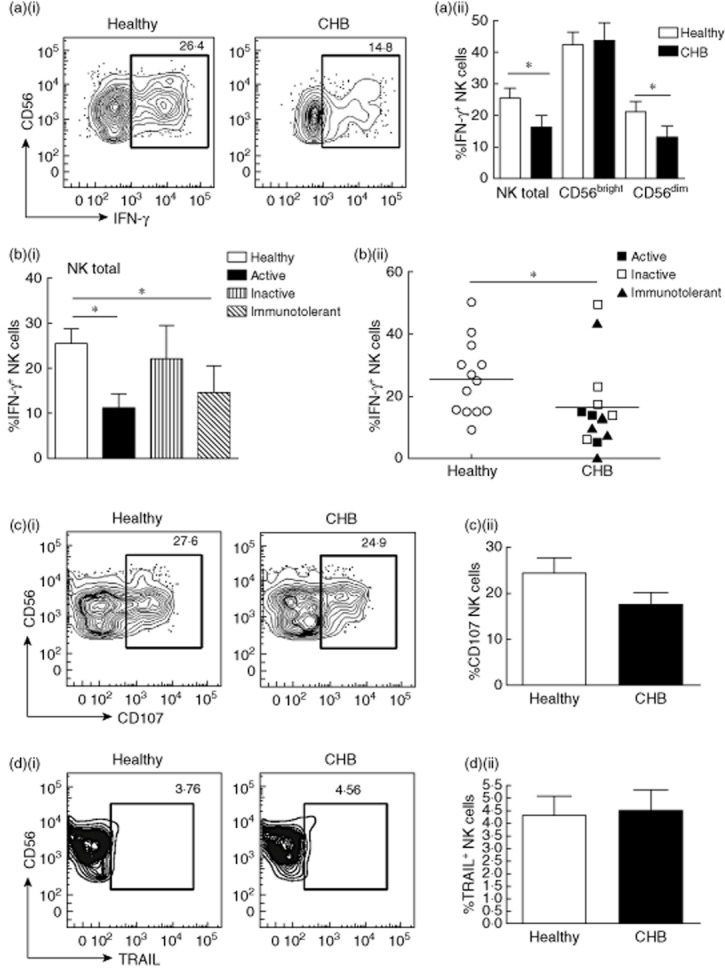
Decreased interferon (IFN)-γ production by natural killer (NK) cells in paediatric chronic hepatitis B (CHB). [a(i)], Representative contour plots from a healthy child and a CHB child showing NK cell IFN-γ production and [a(ii)] summary bar charts comparing production of IFN-γ from total NK cells, CD56 ^bright^ and CD56^dim^ NK cell subsets in children with CHB (*n* = 14, black bars) and healthy children (*n* = 13, white bars). [b(i)], Summary bar charts of IFN-γ-producing total NK cells, CD56^bright^ and CD56^dim^ NK cells in children with CHB in different disease phases, active (*n* = 3), inactive (*n* = 5) and immunotolerant (*n* = 6), and healthy children (*n* = 13). [b(ii)], Each dot plot compares the amount of NK cells expressing IFN-γ in healthy children (o) and CHB children classified as active (■), inactive (□) or immunotolerant (▲). Horizontal lines indicate the median percentages. [c(i)], Representative contour plots from a healthy child and a CHB child showing CD107 expression on NK cells and [c(ii)] summary bar charts comparing CD107 expression on NK cells in children with CHB (*n* = 14) and healthy children (*n* = 13). [d(i)], Representative contour plots from a healthy child and a child with CHB showing NK cell tumour necrosis factor related apoptosis-inducing ligand (TRAIL) expression and [d(ii)], summary data of NK cell TRAIL expression from 15 CHB children and 13 healthy children. Bars represent the mean ± standard error of the mean.*P < 0·05.

NK cells are a potent source of cytokines such as IFN-γ that, in addition to its potential proinflammatory effects, can mediate important direct non-cytolytic anti-viral effects on HBV 30,31. Stimulation of PBMCs from children with CHB with a combination of IL-12/18 showed a 1·5-fold reduction in IFN-γ-producing NK cells compared to healthy controls [Fig. 2a(i,ii)]. Although the CD56^bright^ subset was thought originally to be the main cytokine-producing subset, it is now recognized that the CD56^dim^ subset makes a significant contribution to IFN-γ secretion 32. Our results indicated reduced IFN-γ in total NK cells derived from the CD56^dim^ subset, whereas production of IFN-γ was maintained from the CD56^bright^ subset [Fig. 2a(ii)]. The reduced capacity of NK cells to produce IFN-γ was most notable in patients with liver inflammation and/or high viral load (active, and immunotolerant versus inactive phases, as defined in Table 1) [Fig. 2b(i)]. Interestingly, not all immunotolerant patients were deficient in their ability to produce IFN-γ [Fig. 2b(ii)].

Intracellular TNF-α was not detected after overnight stimulation with IL-12/IL-18 (own observations and reported previously). The ability of NK cells to produce TNF-α was assessed following stimulation with PMA/I, with no significant difference between children with CHB and healthy children (Supporting information, Fig. S1a). PMA/I stimulated cells from patients resulted in similar levels of IFN-γ production compared to healthy controls, suggesting that NK cells retain their ability to respond to this strong stimulus (Supporting information, Fig. S1b).

In addition to their non-cytolytic ability, NK cells typically kill target cells through cytolytic pathways, including the TRAIL pathway. We have previously found TRAIL to be up-regulated on NK cells during episodes of HBV-related liver inflammation 19,20, contributing to hepatocyte apoptosis during HBV-related liver flares 20, and also limiting HBV-specific T cell responses 16. We therefore screened NK cells for changes in TRAIL expression between CHB and healthy children, and determined their degranulation potential upon co-culture with K562 target cells [major histocompatibility complex (MHC) devoid]. Neither the inducible expression of CD107a nor the *ex-vivo* expression level of TRAIL differed between the groups, demonstrating intact NK cell cytolytic function [Fig. 2c(i)(ii),d(i)(ii)].

### Reduced frequency of NK cells expressing activating natural cytotoxicity receptors in CHB children

To investigate whether the impaired ability of NK cells to produce IFN-γ is paralleled by an altered phenotypical NK cell profile in children with CHB, we performed multi-colour flow cytometry for a panel of activating and inhibitory NK cell receptors. Variation of the NK cell phenotype has been reported with age, highlighting a continuum of changes during immunological ageing 23,24,33.

**Fig 3:**
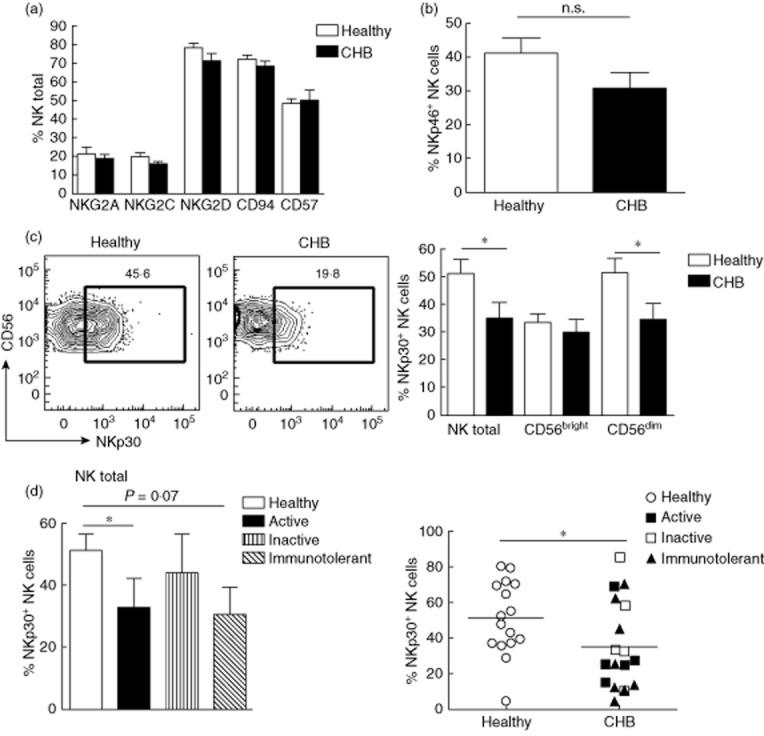
Comparable C-lectin receptor expression and altered natural killer (NK) cell natural cytotoxicity receptor (NCR) expression in chronic hepatitis B (CHB) children. (a) Comparison of the frequency of C-type lectin receptor expression in children with CHB (*n* = 18, black bars) and healthy children (*n* = 16, white bars) and CD57 in children with CHB (*n* = 10, black bars) and healthy children (*n* = 8, white bars). (b) Summary bar charts of expression of natural killer (NK)p46 on NK cells in children with CHB (*n* = 18) and healthy children (*n* = 16). (c) Representative contour plots from a healthy child and a child with CHB demonstrating NK cell NKp30 expression. Bar charts comparing expression of NKp30 on total NK cells, CD56 ^bright^ and CD56^dim^ NK cells in children with CHB (*n* = 18) and healthy children (*n* = 16). (d) Comparison of the frequency of NKp30 expression on total NK cells, CD56^bright^ and CD56^dim^ NK cells in children with CHB classified as active (*n* = 5), inactive (*n* = 5), immunotolerant (*n* = 8) and healthy children (*n* = 18). Each dot plot compares the amount of NKp30 expression on NK cells in healthy children (o), and CHB children classified as active (■), inactive (□) or immunotolerant (▲). Horizontal lines indicate the median percentages. *P < 0·05.

The C-lectin family of receptors are expressed on the surface of NK cells as heterodimers with CD94. They include the CD94/NKG2C activating complex and the inhibitory CD94/NKG2A receptor. Altered expression of these receptors has been described in adult CHB 18,34. However, we found that the frequencies of NK cells expressing the inhibitory receptor NKG2A and the activating receptor NKG2C and co-receptor CD94 were not different between the two groups of children (Fig. 3a, summary data). The levels expressed as mean fluorescent intensity (MFI) were also similar in CHB children and healthy controls (data not shown). Subanalysis of human CD56^+^CD3^–^ NK cell subsets by CD94 surface expression did not reveal any differences between healthy controls and CHB children (data not shown). CMV infection selectively shapes NK cell receptor repertoire in healthy individuals, inducing the expression of NKG2C 35; this phenotypical feature is particularly pronounced in individuals with viral excretion 36. CMV-associated expansion of NKG2C^+^ NK cells has also been reported in adult patients with chronic HBV and hepatitis C virus (HCV) infection 37; however, the levels of expression were highly heterogeneous. In our study, data on CMV seropositivity were not available for healthy children, whereas CHB children were all CMV-seropositive with the exception of one patient, precluding a more detailed analysis of any imprint of CMV on NK cells from infected *versus* healthy children. Interestingly, CMV seropositivity in the study group did not affect NK cell expression of NKG2C or NKG2A. Expression of the activating receptor NKG2D was also found to be similar within the two groups (Fig. 3a).

To further exclude any potential bias conferred by CMV seropositivity on the NK cell repertoire, we analysed the expression of CD57, an important marker of NK cell terminal differentiation and CMV status 38. We detected no significant differences in the expression of CD57 between healthy controls and HBV-infected children (Fig. 3a).

The natural cytotoxicity receptors (NCRs) are a family of activating receptors that are expressed almost exclusively by NK cells and include NKp30, NKp46 and NKp44 39. Analysis of the NCRs demonstrated a trend towards a decreased frequency of NKp46 expressed by NK cells in children with CHB (Fig. 3b), but this did not reach statistical significance. However, expression of NKp30 was reduced on total NK cells of children with CHB [Fig. 3c]. Notably only the CD56^dim^ subset showed significantly decreased expression of NKp30 [Fig. 3c], in line with the restriction of functional defects to this subset. The reduction in the levels of NKp30 expression was more pronounced in children with active disease [Fig. 3d] and varied within immunotolerant patients [Fig. 3d]. Evaluation of NKp30 on total NK cells by MFI also revealed significantly lower values in CHB children (mean ± s.e.m., healthy 640·3 ± 76·27 *versus* CHB 428 ± 90·39 *P* = 0·0218, data not shown). We did not detect any gender-related differences in NKp30+NK cells within and between healthy and CHB children.

### The levels of IL-10 are not increased in children with CHB

The intensity and quality of NK cell cytotoxic and cytokine responses depends further on the cytokine microenvironment. We have demonstrated previously that IL-10 restrains NK cell IFN-γ production in adult patients with CHB, with levels of IL-10 increased significantly in adults with CHB compared to healthy controls 19,40. We therefore examined the cytokine profile in plasma samples from our cohort of CHB children and healthy controls (Table 2). No significant differences were detected in the circulating levels of the panel of 14 plasma cytokines/chemokines examined. In particular, unlike in adults with CHB, the impaired capacity of NK cells to produce IFN-γ was not associated with an increase in circulating IL-10 in paediatric CHB (Table 2).

**Table 2:** Mean value of cytokine concentration ± standard error of the mean in children with chronic hepatitis B (CHB) and healthy controls

	CHB patients (*n* = 18)	Healthy controls (*n* = 16)	*P*-value
IFN-γ	162 ± 87	170 ± 103	0·98
IFN-α	86 ± 42	57 ± 30	0·56
TNF-α	315 ± 312	1003 ± 685	0·83
TNF-β	1424 ± 574	1391 ± 873	0·75
IL-10	13 ± 5	6 ± 3	0·56
IL-12	195 ± 148	15 ± 11	0·57
IL-8	4620 ± 1842	3349 ± 1936	0·75
IL-6	8 ± 2	6 ± 2	0·55
IL-4	0	373 ± 364	0·13
IL-5	1063 ± 1063	2167 ± 1665	0·51
IL-2	112 ± 52	59 ± 34	0·62
IL-1β	347 ± 201	919 ± 644	0·73
CCL3	448 ± 272	736 ± 414	0·74
CXCL9	54 ± 14	223 ± 123	0·42
CXCL10	138 ± 39	84 ± 21	0·34

IFN = interferon; TNF = tumour necrosis factor; IL = interleukin; CCL = C-C chemokine ligand; CXCL = chemokine (C-X-C motif) ligand.

## Discussion

Innate immunity is thought be a major contributor to viral elimination during the early years of life before the adaptive immune system matures 25,41. Accumulating evidence supports a role for NK cells in the immunopathogenesis of CHB in adults 42. In particular, recent data from adults with CHB have highlighted that NK cells are capable of mediating both anti-viral and immunoregulatory functions, while also contributing to hepatocyte turnover and immunopathology via death receptor pathways 16,20,43. However, our knowledge of NK cell function in paediatric populations is limited. Human immunodeficiency virus (HIV) infection is known to alter the phenotype and function of NK cells in children 44. Here we studied the impact of paediatric HBV infection on NK cell phenotype and function in relation to clinical status. Our results indicate that NK cell effector capacity is compromised in HBV-infected children relative to their uninfected counterparts, demonstrating early defects in anti-viral function.

IFN-γ is one of the most prominent cytokines released by NK cells and a potent non-cytolytic mechanism of viral clearance from the HBV-infected liver 45. Our analysis of NK cell effector potential in our paediatric cohort of patients with CHB revealed preservation of cytolytic function and a decrease in IFN-γ production compared to healthy children. The observed defect was more marked in children with active disease. Our results demonstrated that even some children classified as ‘immunotolerant’ retain the ability to respond to cytokine stimuli and produce equivalent levels of cytokines compared to healthy controls, arguing against a state of complete immunological tolerance. The overall NK cell IFN-γ dysfunction was not as extensive as in the adult population with CHB, only affecting the CD56^dim^ subset. By contrast, we found significant reductions in IFN-γ-producing capacity by both CD56^dim^ and CD56^bright^ NK cells in adults with CHB 19, suggesting that cumulative exposure to HBV has a more profound and progressive effect on NK cell anti-viral potential. Thus a pathway specific to cytokine production by CD56^dim^ NK cells is dysregulated in children, similar to adult patients with CHB. However, HBV-infected children retain their ability to respond to stimulation with PMA/I, whereas this effect is lost in the adult population 19. These findings suggest sustained exposure to HBV infection is progressively detrimental to NK cell subset function, reminiscent of the hierarchical loss of effector function exhibited by exhausted T cells. Potent anti-viral therapy in CHB adults partially restores the ability of NK cells to produce IFN-γ from the CD56^dim^ subset and improves their *de-novo* activation 18,19; our data raise the possibility that these defects may be more effectively reversed in paediatric patients by earlier anti-viral treatment.

The observed differences in NK cell function could not be attributed to changes in the proportions of NK cells or subsets. We therefore postulated that NK cells with unique receptor profiles may predominate in paediatric CHB that may partly explain their differences in effector function. The activating NCR NKp30 was significantly down-regulated in CHB children. Importantly, we found that this phenotypical alteration was more prominent in children with active disease and in the CD56^dim^ NK cell subset, analogous to the functional defect, and was not influenced by discrepancies in age and gender within/between the two groups. NKp30 plays an important role in NK–dendritic cell (DC) cross-talk 46 and has also been reported to be down-regulated significantly in adult CHB 18, which may compromise NK–DC interactions. In addition to its ability to induce cytotoxicity, engagement of NKp30 can mediate the production of cytokines such as IFN-γ. In HIV infection, defective interaction with mDC through impaired function of NK cell NKp30 leads to their impaired secretion of IFN-γ by NK cells 47. In adult CHB, mDC are markedly impaired in their ability to activate NK cells, which leads in turn to diminished NK cell IFN-γ production without affecting cytotoxicity 48. Of relevance, the immunoregulatory effects of HBsAg, HBV (whole virion) and HBeAg may impair DC function and thereby further impair NK cell function 49. This may, in turn, influence T cell differentiation and shaping of adaptive immune responses. Equally, defective cross-talk and editing of DCs by NKs could affect the development of adaptive T cell anti-viral immunity through restricting antigen presentation 50,51. Although our findings suggest a role for accessory cells, the contribution of NKp30–NK cell reciprocal interaction with DC in paediatric HBV infection remains to be established. Alternatively, NK cells expressing NKp30 may be recruited preferentially to the liver, the site of HBV replication. Levels of NKp46, despite a trend towards lower expression, were not reduced significantly in children with HBV. This may be important for the maintenance of cytotoxicity. Study of a larger cohort of patients and matched controls could help dissect out the effect of race- and gender-related variations in the expression of NKp46, in line with recent published associations in the context of hepatitis C virus (HCV) 52. Although we did not analyse the expression of killer cell immunoglobulin-like receptors (KIR) due to limited samples, it is well known that different KIR–HLA combinations can modulate NK cell function and influence the outcome of infectious diseases with HIV and HCV infections 53,54. Further work is required to explore how the KIR repertoire is shaped during the course of CHB and how perinatal infection with HBV influences NK cell licensing. The added effect of CMV and other viral pathogens such as Epstein–Barr Virus (EBV) on shaping of NK cell receptors and maturational status requires further evaluation in a larger cohort of patients and healthy donors of similar ethnicity. As age and time of infection with additional pathogens may be both relevant and inter-related, a prospective longitudinal assessment would be desirable to remove any potential confounding factors on the analysis of the impact of HBV infection. However, the lack of expansion of NK cells expressing NKG2C and CD57 in our cohort of HBV-infected children argues against CMV impacting upon the observed NK cell phenotypical and functional differences in this study.

The cytokine environment can further shape the effector functions of NK cells. In adult CHB elevated levels of IL-10, especially in patients with active disease, selectively modulated NK cell anti-viral function 19. In this study we did not detect high levels of IL-10 in CHB children compared to controls. However, it should be noted that only a small number of children had evidence of liver inflammation. No alterations in the cytokine milieu were detected to account for the observed deficiency in NK cell IFN-γ production in the cross-sectional comparison. Future longitudinal studies could assess the potential impact of disease fluctuations on cytokine composition and NK cell homeostasis, phenotype and effector function.

In summary, we have characterized for the first time a functional defect in NK cell IFN-γ production in paediatric CHB. This defect was more prominent in the group of children with active disease. The progressive decline in IFN-γ production by NK cells seen in adults could represent the cumulative effect of HBV infection on NK cell anti-viral function. Reduced expression of the NCR NKp30 on NK cells could account partially for this deficiency via impaired NK–DC reciprocal interactions. Implementation of universal vaccination against HBV in newborns has already reduced the incidence of HBV-related HCC in countries such as Taiwan 55. For those chronically infected, a remaining challenge is the development of a treatment strategy able to induce HBV immune control, especially in immunotolerant children 9. Thus a more comprehensive understanding of the immune responses during childhood is highly desirable. In adult CHB, anti-viral treatment can partially restore NK cell defects in IFN-γ production. Our data have important implications for the clinical management of paediatric HBV patients. Taken together with recent findings on T cell responses 8, they support consideration of earlier initiation of treatment to include young patients in the immunotolerant phase, in agreement with recent studies highlighting their favourable response to treatment 56,57.

## Supporting Information

**Fig. S1.** Summary bar charts of natural killer (NK) cell tumour necrosis factor (TNF)-α production (a) and NK cell interferon (IFN)-γ production (b) after phorbol myristate acetate (PMA)/I stimulation in healthy children (*n* = 13) and children with chronic hepatitis B virus (CHB) (*n* = 14). Bars represent the mean ± standard error of the mean. Statistical analysis was performed using the Mann–Whitney *U*-test.

## References

[b1] Liaw YF, Chu CM (2009). Hepatitis B virus infection. Lancet.

[b2] McMahon BJ, Alward WL, Hall DB (1985). Acute hepatitis B virus infection: relation of age to the clinical expression of disease and subsequent development of the carrier state. J Infect Dis.

[b3] Lok AS, McMahon BJ (2009). Chronic hepatitis B: update 2009. Hepatology.

[b4] McMahon BJ (2009). The natural history of chronic hepatitis B virus infection. Hepatology.

[b5] Shimakawa Y, Yan HJ, Tsuchiya N, Bottomley C, Hall AJ (2013). Association of early age at establishment of chronic hepatitis B infection with persistent viral replication, liver cirrhosis and hepatocellular carcinoma: a systematic review. PLOS ONE.

[b6] Ni YH (2011). Natural history of hepatitis B virus infection: pediatric perspective. J Gastroenterol.

[b7] Ganem D, Prince AM (2004). Hepatitis B virus infection – natural history and clinical consequences. N Engl J Med.

[b8] Kennedy PT, Sandalova E, Jo J (2012). Preserved T-cell function in children and young adults with immune-tolerant chronic hepatitis B. Gastroenterology.

[b9] Chang MH (2013). Paediatrics: children need optimal management of chronic hepatitis B. Nat Rev Gastroenterol Hepatol.

[b10] Vivier E, Tomasello E, Baratin M, Walzer T, Ugolini S (2008). Functions of natural killer cells. Nat Immunol.

[b11] Vivier E, Raulet DH, Moretta A (2011). Innate or adaptive immunity? The example of natural killer cells. Science.

[b12] Narni-Mancinelli E, Ugolini S, Vivier E (2013). Tuning the threshold of natural killer cell responses. Curr Opin Immunol.

[b13] Crome SQ, Lang PA, Lang KS, Ohashi PS (2013). Natural killer cells regulate diverse T cell responses. Trends Immunol.

[b14] Waggoner SN, Cornberg M, Selin LK, Welsh RM (2012). Natural killer cells act as rheostats modulating antiviral T cells. Nature.

[b15] Lang PA, Lang KS, Xu HC (2012). Natural killer cell activation enhances immune pathology and promotes chronic infection by limiting CD8+ T-cell immunity. Proc Natl Acad Sci USA.

[b16] Peppa D, Gill US, Reynolds G (2013). Up-regulation of a death receptor renders antiviral T cells susceptible to NK cell-mediated deletion. J Exp Med.

[b17] Oliviero B, Varchetta S, Paudice E (2009). Natural killer cell functional dichotomy in chronic hepatitis B and chronic hepatitis C virus infections. Gastroenterology.

[b18] Tjwa ET, van Oord GW, Hegmans JP, Janssen HL, Woltman AM (2011). Viral load reduction improves activation and function of natural killer cells in patients with chronic hepatitis B. J Hepatol.

[b19] Peppa D, Micco L, Javaid A (2010). Blockade of immunosuppressive cytokines restores NK cell antiviral function in chronic hepatitis B virus infection. PLOS Pathog.

[b20] Dunn C, Brunetto M, Reynolds G (2007). Cytokines induced during chronic hepatitis B virus infection promote a pathway for NK cell-mediated liver damage. J Exp Med.

[b21] Micco L, Peppa D, Loggi E (2013). Differential boosting of innate and adaptive antiviral responses during pegylated-interferon-alpha therapy of chronic hepatitis B. J Hepatol.

[b22] Dominguez E, Madrigal JA, Layrisse Z, Cohen SB (1998). Fetal natural killer cell function is suppressed. Immunology.

[b23] Le Garff-Tavernier M, Beziat V, Decocq J (2010). Human NK cells display major phenotypic and functional changes over the life span. Aging Cell.

[b24] Almeida-Oliveira A, Smith-Carvalho M, Porto LC (2011). Age-related changes in natural killer cell receptors from childhood through old age. Hum Immunol.

[b25] Lee YC, Lin SJ (2013). Neonatal natural killer cell function: relevance to antiviral immune defense. Clin Dev Immunol.

[b26] Dunn C, Peppa D, Khanna P (2009). Temporal analysis of early immune responses in patients with acute hepatitis B virus infection. Gastroenterology.

[b27] Sundstrom Y, Nilsson C, Lilja G, Karre K, Troye-Blomberg M, Berg L (2007). The expression of human natural killer cell receptors in early life. Scand J Immunol.

[b28] Almehmadi M, Flanagan BF, Khan N, Alomar S, Christmas SE (2014). Increased numbers and functional activity of CD56(+) T cells in healthy cytomegalovirus positive subjects. Immunology.

[b29] Ferlazzo G, Morandi B, D'Agostino A (2003). The interaction between NK cells and dendritic cells in bacterial infections results in rapid induction of NK cell activation and in the lysis of uninfected dendritic cells. Eur J Immunol.

[b30] Guidotti LG, Ishikawa T, Hobbs MV, Matzke B, Schreiber R, Chisari FV (1996). Intracellular inactivation of the hepatitis B virus by cytotoxic T lymphocytes. Immunity.

[b31] Kakimi K, Guidotti LG, Koezuka Y, Chisari FV (2000). Natural killer T cell activation inhibits hepatitis B virus replication *in vivo*. J Exp Med.

[b32] Fauriat C, Long EO, Ljunggren HG, Bryceson YT (2010). Regulation of human NK-cell cytokine and chemokine production by target cell recognition. Blood.

[b33] Lutz CT, Moore MB, Bradley S, Shelton BJ, Lutgendorf SK (2005). Reciprocal age related change in natural killer cell receptors for MHC class I. Mech Ageing Dev.

[b34] Bonorino P, Ramzan M, Camous X (2009). Fine characterization of intrahepatic NK cells expressing natural killer receptors in chronic hepatitis B and C. J Hepatol.

[b35] Guma M, Angulo A, Vilches C, Gomez-Lozano N, Malats N, Lopez-Botet M (2004). Imprint of human cytomegalovirus infection on the NK cell receptor repertoire. Blood.

[b36] Monsivais-Urenda A, Noyola-Cherpitel D, Hernandez-Salinas A (2010). Influence of human cytomegalovirus infection on the NK cell receptor repertoire in children. Eur J Immunol.

[b37] Beziat V, Dalgard O, Asselah T (2012). CMV drives clonal expansion of NKG2C+ NK cells expressing self-specific KIRs in chronic hepatitis patients. Eur J Immunol.

[b38] Nielsen CM, White MJ, Goodier MR, Riley EM (2013). Functional significance of CD57 expression on human NK cells and relevance to disease. Front Immunol.

[b39] Moretta L, Moretta A (2004). Unravelling natural killer cell function: triggering and inhibitory human NK receptors. EMBO J.

[b40] Das A, Ellis G, Pallant C (2012). IL-10-producing regulatory B cells in the pathogenesis of chronic hepatitis B virus infection. J Immunol.

[b41] Orange JS, Ballas ZK (2006). Natural killer cells in human health and disease. Clin Immunol.

[b42] Rehermann B (2013). Pathogenesis of chronic viral hepatitis: differential roles of T cells and NK cells. Nat Med.

[b43] Maini MK, Peppa D (2013). NK cells: a double-edged sword in chronic hepatitis B virus infection. Front Immunol.

[b44] Ballan WM, Vu BA, Long BR (2007). Natural killer cells in perinatally HIV-1-infected children exhibit less degranulation compared to HIV-1-exposed uninfected children and their expression of KIR2DL3, NKG2C, and NKp46 correlates with disease severity. J Immunol.

[b45] Guidotti LG, Rochford R, Chung J, Shapiro M, Purcell R, Chisari FV (1999). Viral clearance without destruction of infected cells during acute HBV infection. Science.

[b46] Ferlazzo G, Tsang ML, Moretta L, Melioli G, Steinman RM, Munz C (2002). Human dendritic cells activate resting natural killer (NK) cells and are recognized via the NKp30 receptor by activated NK cells. J Exp Med.

[b47] Mavilio D, Lombardo G, Kinter A (2006). Characterization of the defective interaction between a subset of natural killer cells and dendritic cells in HIV-1 infection. J Exp Med.

[b48] Shi CC, Tjwa ET, Biesta PJ (2012). Hepatitis B virus suppresses the functional interaction between natural killer cells and plasmacytoid dendritic cells. J Viral Hepat.

[b49] Woltman AM, Op den Brouw ML, Biesta PJ, Shi CC, Janssen HL (2011). Hepatitis B virus lacks immune activating capacity, but actively inhibits plasmacytoid dendritic cell function. PLOS ONE.

[b50] Moretta A (2002). Natural killer cells and dendritic cells: rendezvous in abused tissues. Nat Rev Immunol.

[b51] Walzer T, Dalod M, Robbins SH, Zitvogel L, Vivier E (2005). Natural-killer cells and dendritic cells: ‘l'union fait la force’. Blood.

[b52] Golden-Mason L, Stone AE, Bambha KM, Cheng L, Rosen HR (2012). Race- and gender-related variation in natural killer p46 expression associated with differential anti-hepatitis C virus immunity. Hepatology.

[b53] Khakoo SI, Thio CL, Martin MP (2004). HLA and NK cell inhibitory receptor genes in resolving hepatitis C virus infection. Science.

[b54] Martin MP, Qi Y, Gao X (2007). Innate partnership of HLA-B and KIR3DL1 subtypes against HIV-1. Nat Genet.

[b55] Ni YH, Chen DS (2010). Hepatitis B vaccination in children: the Taiwan experience. Pathol Biol (Paris).

[b56] D'Antiga L, Aw M, Atkins M, Moorat A, Vergani D, Mieli-Vergani G (2006). Combined lamivudine/interferon-alpha treatment in ‘immunotolerant’ children perinatally infected with hepatitis B: a pilot study. J Pediatr.

[b57] Carey I, D'Antiga L, Bansal S (2011). Immune and viral profile from tolerance to hepatitis B surface antigen clearance: a longitudinal study of vertically hepatitis B virus-infected children on combined therapy. J Virol.

