# Supplementary figures and images for: Defective natural killer cell anti-viral capacity in paediatric HBV infection
Source: Clin Exp Immunol. 2015 Feb 16;179(3):466–76. doi: 10.1111/cei.12470 (PMC4337679; doi:10.1111/cei.12470)

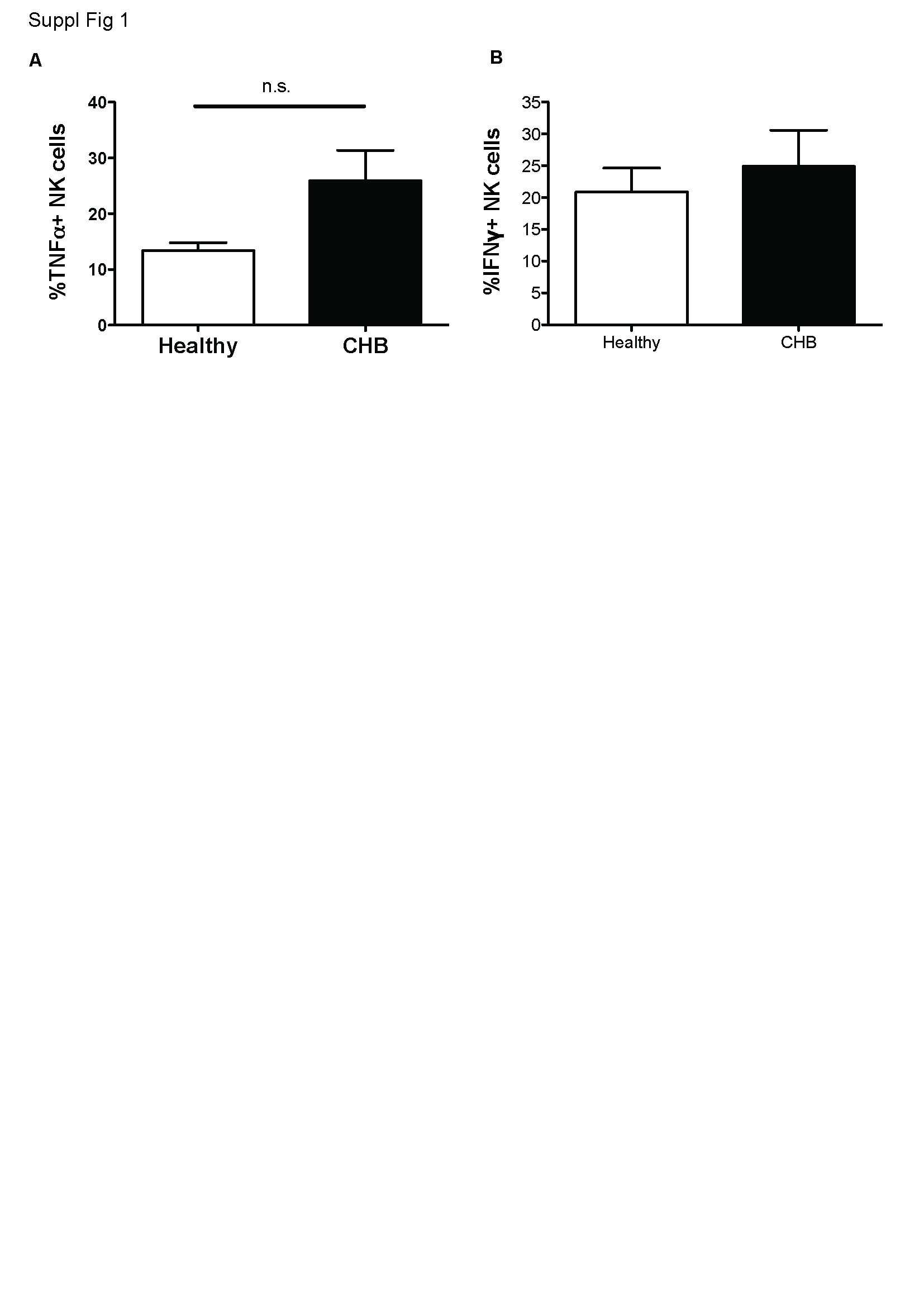

Supplement: Supplementary file 1 [file cei0179-0466-sd1.tif]
